# Girls start life on an uneven playing field: Evidence from lowland rural Nepal

**DOI:** 10.1093/emph/eoac029

**Published:** 2022-08-04

**Authors:** Akanksha A Marphatia, Naomi S Saville, Dharma S Manandhar, Mario Cortina-Borja, Alice M Reid, Jonathan C K Wells

**Affiliations:** Department of Geography, University of Cambridge, Cambridge CB2 3EN, UK; Population, Policy and Practice Research and Teaching Department, UCL Great Ormond Street Institute of Child Health, London WC1N 1EH, UK; Institute for Global Health, University College London, London WC1N 1EH, UK; Mother and Infant Research Activities, Kathmandu, Nepal; Population, Policy and Practice Research and Teaching Department, UCL Great Ormond Street Institute of Child Health, London WC1N 1EH, UK; Department of Geography, University of Cambridge, Cambridge CB2 3EN, UK; Population, Policy and Practice Research and Teaching Department, UCL Great Ormond Street Institute of Child Health, London WC1N 1EH, UK

## Abstract

**Background and objectives:**

Evolutionary research on the sex ratio at birth (SRB) has focused on explaining variability within and between populations, and whether parental fitness is maximized by producing daughters or sons. We tested predictors of SRB in a low-income setting, to understand whether girls differ from boys in their likelihood of being born into families with the capacity to invest in them, which has implications for their future health and fitness.

**Methodology:**

We used data from a cluster randomized control trial from lowland rural Nepal (16 115 mother-child dyads). We applied principal component analysis to extract two composite indices reflecting maternal socio-economic and reproductive (parity, age) capital. We fitted mixed-effects logistic regression models to estimate odds ratios of having a girl in association with these individual factors and indices.

**Results:**

The SRB was 112. Compared to the global reference SRB (105), there were seven missing girls per 100 boys. Uneducated, early-marrying, poorer and shorter mothers were more likely to give birth to girls. Analysing composite maternal indices, lower socio-economic and reproductive capital were independently associated with a greater likelihood of having a girl.

**Conclusions and implications:**

In this population, girls start life facing composite disadvantages, being more likely than boys to be born to mothers with lower socio-economic status and reproductive capital. Both physiological and behavioural mechanisms may contribute to these epidemiological associations. Differential early exposure by sex to maternal factors may underpin intergenerational cycles of gender inequality, mediated by developmental trajectory, education and socio-economic status.

## INTRODUCTION

Globally, the average sex ratio at birth (SRB) is 107 males per 100 female births [1]. However, this is skewed by higher SRBs in countries with strong son preferences and access to pre-natal sex selection. For these reasons, the United Nations (UN) uses 105:100 as the ‘expected biological sex ratio’ at birth [2] and we follow this approach in our analysis described below. The slight male bias has been attributed to sex differences in the pattern of mortality during pregnancy: the sex ratio appears equal at conception, but overall, female mortality is slightly higher than male mortality over the early stages of gestation, resulting in a slight excess of males by birth, despite higher male mortality towards the end of gestation and during the process of birth [3]. In South Asia, more boys than girls are born each year compared to UN’s expected SRB. For example, data from 2017 indicate an SRB of 110:100 for India and 107:100 for Nepal, where our study is based [4], with the UN calculating similar values [1]. However, the SRB within Nepal also varies substantially by region [5].

To explain SRB variability within and between populations, researchers have investigated whether biological traits or parental behaviour patterns can help predict whether individual women are more likely to produce sons or daughters. A systematic review identified 10 factors influencing the SRB, ranging from individual level biological factors, such as maternal-stress and coital rates, to environment-related factors, such as exposure to air pollution from incinerators, access to sex-determination technology and abortion of female foetuses [6]. To frame such analyses, many have drawn explicitly on evolutionary theory, to investigate whether SRB variability may have an adaptive element.

For example, the well-known Trivers–Willard hypothesis [7] proposed that since in mammals reproductive success tends to be more variable in adult males than females, and since adult phenotype bears an imprint of maternal investment in early life, mothers would maximize their fitness if they were more likely to produce sons when in good condition, and daughters if not in good condition. This hypothesis has attracted much attention, both in non-human mammals and humans. There is growing theoretical [8] and mechanistic [9, 10] support for the notion that components of maternal nutritional status can influence the SRB, however, support specifically for the Trivers–Willard hypothesis of SRB variability in humans remains weak and inconsistent [11–14].

An alternative approach focuses less on individual maternal traits, and more on ecological factors impacting the whole population. According to the ‘frail male’ hypothesis, males are considered more vulnerable to external stresses, resulting in changes in the SRB of the population over time in association with varying ecological conditions [15]. In the USA, for example, fewer boys were born alive during the Spanish Flu epidemic and the Great Depression than at other times, whereas individual-level markers of maternal condition did not predict the SRB [15]. Similarly, a study from Nepal found that exposure to adverse conditions during pregnancy, such as civil conflict, were associated with higher mortality of sons *in utero* and hence a higher likelihood of having a daughter [16].

At a broader level, any family factors that contribute to SRB variability merit attention through the lens of gender inequality [17, 18]. If maternal characteristics shape the likelihood of having sons versus daughters, then the two sexes may on average not be born into identical family environments. Moreover, more gender-unequal societies may have a stronger son preference, as found in a study comparing two villages with different levels of women’s autonomy and fertility in Nepal [19]. These issues have attracted relatively little attention in evolutionary studies which have focused on the implications of the SRB for the reproductive fitness of the mother, rather than the direct implications for the offspring.

Our study therefore aims to analyse predictors of the SRB in a low-income setting, in order to understand whether girls and boys start life equally distributed across a population, or whether they are differentially likely to cluster in association with socio-economic, demographic or biological factors that may shape the capacity for families to invest in them. Given compelling evidence that experience in early life has substantial implications for adult health, human capital and reproductive fitness [20–23], the notion that girls and boys might on average pass through different societal and biological ‘niches’ in early life merits attention. By going beyond the Trivers–Willard hypothesis, we aim to offer a more comprehensive perspective on gender inequality and gain new insight into its intergenerational transmission.

At the level of society, there may be several reasons why the SRB is actively skewed towards a male bias, reflecting socio-economic dynamics. For example, in South Asian patrilineal societies where the economic contributions of men are important in maintaining parents in older age, and the household over time, families prioritize investment in raising sons [24]. Moreover, in some societies including the context of our study, the absolute ‘cost’ of raising girls increases through life stages not only for basic care, food and education but also for dowry payments at marriage [25]. In patrilocal societies, investment in girls is considered to be ‘lost’ to the natal family because at marriage a girl moves to her husband’s home, whereas a boy is generally expected to remain in the natal home with his new wife [26]. Analysing data across and within 108 countries, Ebenstein found a strong correlation between a high SRB and countries practicing patrilocality, where a high proportion of parents co-reside with sons and benefit from elder age care from their daughters-in-law, rather than daughters [27]. In the same countries, intensive agriculture is widely practiced, which increases the value of land and hence of its inheritance through the male line [27].

To satisfy any preference for having sons, there may be active and deliberate practices to selectively abort female foetuses using sex-determination technology. In Nepal, direct evidence for this practice is sparse, due to it being illegal and punishable by imprisonment. Nevertheless, it is apparent that the SRB became more skewed after the legalization of abortion in 2002 [28]. Indirect evidence also comes from the finding that approximately one-quarter of the national population lives in urban regions where the SRB is >110, suggesting that up to 10% of girls may be missing in some urban localities [5].

The practice of sex-selective abortion seems to become more prominent for later births [29]. Since males have higher infant and child mortality rates, parents with a strong son preference may want to have more boys to increase the chance of having surviving sons [30]. Several studies have reported that families are more likely to have a boy as the second or third child, if the first- and second-born children were girls [31–34]. Conversely, a national survey of Nepal suggests that since the likelihood of having a son is lower as fertility rates decline, sex-selective abortion is increasingly used by wealthier and more educated groups [5].

Beyond efforts to influence the sex of children born into the family, parents may also invest differently in sons versus daughters after they have been born, in relation to outcomes such as health and education. The extent to which this occurs appears to vary between settings. In the USA, wealthier families do not appear to invest more in sons than daughters [35], whereas Greece and Spain have skewed sex ratios at birth and throughout infancy and childhood [36, 37]. In low-income settings, and especially in South Asia, son-preference is well described [24, 38, 39], and follows the same logic as that described for artificial sex selection *in utero* described above. In this context, gender inequality may be perpetuated after birth. For example, in Nepal, parents invest more in educating sons than daughters, as shown by lower girls’ secondary educational attainment [40, 41]. Whilst during childhood and adolescence boys appear to be more stunted and thinner than girls in Nepal, once girls marry, they tend to eat last and less at meals they prepare in their marital homes, which contributes to their undernutrition [42, 43]. Another study in South Asian countries including Nepal found that son preference interacted with sibling composition with multiple brothers and sisters increasing girls’ risk for acute (wasting) and chronic (stunting/underweight) malnutrition [44].

Using data from a cluster randomized control trial of 16 115 mother–child dyads from lowland rural Nepal, we first determined the SRB in this population. We then identified individual markers of maternal capital, and also developed composite markers that captured the variability. Building on the conceptual model of embodied capital [45], traits that promote the capacity for maternal investment can be considered ‘maternal capital’ [46]. This term brings together diverse somatic, social and socio-economic components of maternal phenotype, which are all markers of the mother’s ability to invest in the offspring [46]. For example, mothers with more social capital may be able to bring together a wider pool of social support for childcare [47], mothers with greater education and empowerment may be able to steer more economic resources to childcare [48–50], and mothers with higher levels of lean and fat mass may be able to invest more through physiological pathways in foetal and infant growth respectively [51]. Using maternal capital markers as the exposure, we tested the overarching hypothesis that mothers with lower levels of capital were more likely to give birth to girls, compared to those with higher capital. We developed hypotheses for specific components of maternal capital as described in the methods section.

## METHODOLOGY

### Study profile

We analyse data from the cluster-randomized controlled Low Birth Weight South Asia Trial (LBWSAT) in rural lowland Nepal (Terai), which assessed the impact of interventions during pregnancy on the birth weight and growth of children from birth to 16 months of age [52]. The trial spanned 80 geographic clusters (Village Development Committees, VDCs) in southern Mahottari and Dhanusha districts bordering Bihar state in India. The Maithili-speaking Madhesi women of our study have the lowest median marriage age (15 years) and educational attainment (about three-fourths have no schooling) in Nepal [53, 54]. Families generally decide when and who girls marry and seclusion norms often restrict women to households, where they have low levels of agency [55, 56].

Of the 63 308 married women consenting to menstrual monitoring, 25 090 women aged 10–49 years fell pregnant and were recruited between December 2013 and February 2015 into one of three interventions: (i) Women’s Groups using the Participatory Learning and Action (PLA) behaviour change approach, (ii) PLA and unconditional cash transfers and (iii) PLA and food supplementation or (iv) a control group accessing Government of Nepal health services [52].

The Nepal Health Research Council (108/2012; 292/2018), University College London (4198/001, 0326/015) and University of Cambridge (1016, the secondary analysis only) granted research ethics approvals for the trial and secondary analysis of LBWSAT data. VDC secretaries consented to the inclusion of clusters. Written consent was obtained from women and guardians of married adolescents aged <18 years.

### Data

Women’s age and their age at marriage were recorded in running years and converted to completed years (running years minus 1). Our outcome variable was sex of the child born into the LBWSAT, and our interest was in identifying the factors associated with having a ‘girl’. Exposures relating to maternal phenotype included:

Education: none, primary (1–5 years) or lower-secondary/higher (≥6 years);
Marriage age: ≤14 years, 15 years, 16–17 years or ≥18 years;
Parity: 0, 1, 2 or ≥3 births; and
Height: ≤144.9 cm, 145–154.9 cm or ≥155 cm.

Education was coded according to the Nepali education system [57]. In regression analyses, marriage was coded in two groups, below and above the median age of 15 years in our study population. Height was coded according to established cut-offs for this population for short stature [58], and our own cut-offs for taller stature.

Exposures relating to socio-economic characteristics included:

Caste: disadvantaged Muslim, disadvantaged Dalit, Middle (Janjati, Terai castes) or advantaged (Yadav, Brahmin);
Assets: quartiles, with 1 being the poorest and 4 the richest;
Land-holding: none, ≤0.5 hectares, 0.51–0.99 hectares or ≥1 hectare; and
Accessibility to bazaar: ≤30 , 31–89 or ≥90 minutes.

The asset score, land-holding and bazaar variables were coded according to the distribution in our data. Accessibility to the nearest big bazaar (with fixed stores) was measured in minutes using the usual form of transport.

The asset score was derived using principal component analysis (PCA). The first principal component had positive factor loadings for all 11 variables and was taken as the marker of material wealth. It accounted for 31.2% of the variability, compared to 10.9% and 9.7% from the second and third principal components. Variables contributing the highest factor loadings (weight) to the first principal component included: wall material (0.393), toilet type (0.382), roof material (0.382), flooring material (0.371), motorbike (0.311), number of rooms used for sleeping (0.302), television (0.272), access to electricity (0.237), drinking water source (0.209), non-biomass cooking fuel use (0.174) and computer (0.157). Land-holding was excluded from the asset score because we wanted to test its association with having a girl, independent of material wealth.

PCA, described below, was also used to derive two composite indices of maternal capital. The two components that emerged from this approach captured different axes of variability in maternal capital. One component is related to socio-economic resources, and the other to reproductive capital, as explained in more detail below. We then investigated their independent association with the odds of having a girl.

### Hypotheses

We first use these data to test the following specific predictions:

1. that mother’s lower stature, low education and early marriage age are independently associated with having a relatively high proportion of daughters;
2. that independent of maternal phenotype, low paternal education, low household wealth and disadvantaged caste group are associated with having a relatively high proportion of daughters; and
3. that easier accessibility to the nearest ‘big market’ (a proxy for access to technology for sex-specific abortions) is associated with a lower likelihood of having daughters.

We then explore two composite indices to get an overview of the independent associations of maternal socio-economic and reproductive capital components.

### Statistical methods

We summarized women’s age and age at marriage with median and interquartile range given their skewed distributions. We used chi-squared tests (categorical variables) and non-parametric k-sample analysis of variance (Kruskal–Wallis test; continuous variables) to test (i) for differences in maternal and household traits by child’s sex, (ii) for bias in these characteristics where child sex was missing and (iii) for bias in child sex where key predictor variables were missing.

Data were scaled to unit variance before performing the PCA indexing socio-economic capital and maternal reproductive capital (confirmed by biplot, Supplementary Fig. S1). The first composite score derived from PCA explained 33.7% of variability. Variables contributing the highest factor loading (weight) included: maternal education (0.776), husband’s education (0.733), household assets (0.620), land-holding (0.481) and caste (0.462). As these individual variables all related to socio-economic traits, we refer to this composite marker as ‘socio-economic capital’. The second composite score explained 19.1% of variability. The variables contributing the highest factor loading (weight) were: maternal parity (0.740) and maternal age (0.719). Since later maternal age at childbirth and greater parity have both been associated with a greater birth weight of the offspring in this population [59], we refer to this composite marker as ‘reproductive capital’. Maternal height was not included in these indices because it would reduce our sample size, and, when included in the PCA on the dataset including available maternal height data it had a relatively low factor loading. In each case, the continuous composite scores were coded into quartiles, indicating low to high (1–4, respectively) levels of maternal socio-economic and reproductive capital.

A heatmap compared LBWSAT SRBs for the two composite indices relative to the global population SRB by adjusting for the UN-recognized average value, i.e. (105-LBWSAT SRB). We fitted mixed-effects logistic regression models with a random effect on the intercept accounting for within-cluster variability. We estimated adjusted odds ratios of having a girl with all of the maternal and household characteristics together, and by the two composite maternal capital indices. Models controlled for parity and maternal age. Goodness-of-fit was evaluated by the Nakagawa–Schielzeth conditional *R*^2^ value, which measures the percentage of variance explained by the model’s fixed and random effects [60]. The higher value of each trait was set as the reference group for exposure variables (e.g. richest households). For accessibility to big bazaar, the shortest time was set as the reference.

Models adjusted for trial arm, but as these associations were not statistically significant, they were not reported in tables. As the trial recruited pregnant married women, interventions could not have influenced women’s marriage age, education (which typically ends before/at marriage) or husband’s education and caste.

The SRB was calculated in Microsoft Excel (version 16.16.23). Statistical analyses were conducted in R version 4.04 [61] and RStudio version 1.4.1106 using the R libraries tidyverse [62] and lme4 [63] (mixed-effects regression models). The R library FactoMineR [64] was used to compute the two PCA composite ‘maternal capital’ indices and the PCA biplot.

## RESULTS

### Sample selection

**Figure 1.**
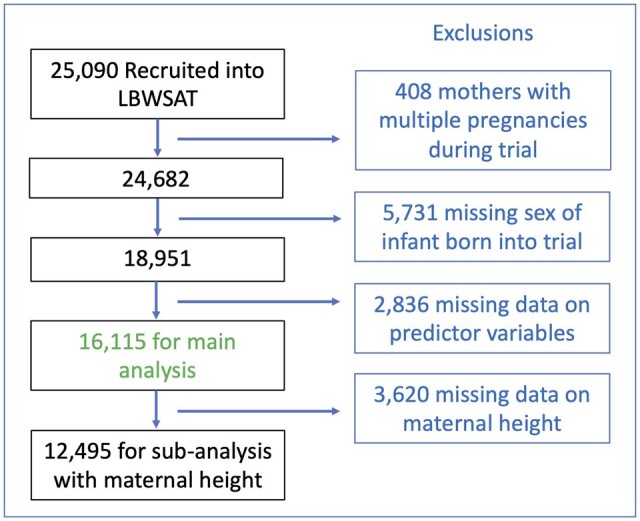
Sample selection. The flowchart illustrates our sample selection. It shows which women were excluded, and the sample size used in the main analysis and subset of women with data on height

Our main analysis comprised of 16 115 women. The analysis including maternal stature included a subset of 12 495 women with data on height. Of 25 090 married pregnant women recruited into the LBWSAT, we excluded 408 mothers with multiple pregnancies during the trial, 5731 mothers missing data on the outcome variable (sex of the infant born into the trial) and 2836 mothers missing data on exposures variables (Fig. 1).

There were two factors that reduced the availability of data on offspring sex [65]. First, several thousand women (*n* = 5410) were recruited into the trial before the intervention began, or when the intervention was still being ‘run in’. These women delivered before birth data capture began. Second, the entire trial was brought to an end prematurely, and birth data capture was therefore halted before many women had delivered. Although many participants who missed birth data capture for these two reasons were subsequently captured through a ‘trial endpoint’ measurement, undertaken during infancy, the records show that 5731 recruited women never attended any follow-up after delivery, and hence the sex of the baby was not ascertained.

Compared to mothers with data on the sex of infant born into the trial, mothers with missing data on this variable showed trends to lower maternal age, greater education, lower parity, advantaged caste, greater household assets and large land (Supplementary Table S1). However, the magnitudes of these differences were trivial and therefore not expected to bias our results. There was no bias in child sex between the sample used in our analysis and those excluded for missing data on predictor variables (Supplementary Table S2).

### Sample description

Of the 16 115 women in our sample, 52.8% (*n* = 8501) gave birth to a boy and 47.2% (*n* = 7614) to a girl. The SRB for the sample was thus 112. Compared to the global population SRB of 105, there were an additional 7 missing girls relative to 100 boys in our study. Simulations indicated that the SRB of the population with missing sex data (*n* = 5731) would need to be 84:100, in order to bring the SRB for the whole sample back to 105:100 (Supplementary Fig. S2).

Mothers’ median age was 21 years and 65% were uneducated compared to 49% of husbands (Table 1). Only 10% of women had married ≥18 years, with 35% ≤14 years, 26% at 15 years and 29% between 16 and 17 years. About 15% of women were of shorter stature, 65% of average height and 20% were tall. More than half of the mothers were of parity 0 or 1. About 35% of households were from the disadvantaged caste, 43% and 22% from the middle and advantaged castes respectively. Household assets were presented in quartiles. About 36% of the households owned no land, 32% ≤0.5 hectares, 47% between 0.51 and 0.99 hectares and 17% ≥1 hectare. Big bazaars were easily accessible to about half of the families, 41% lived 31–89 min away and 9% ≥90 min away.

**Table 1.** Characteristics of sample and differences by sex of infant born into LBWSAT

	**Full sample**		**Boys**		**Girls**		
	**(*n *=* *16** **115)**		**(*n *=* *8501)**		**(*n *=* *7614)**		
	**Median**	**IQR**	**Median**	**IQR**	**Median**	**IQR**	
**Women’s age (years)**	21	6	21	6	21	6	
	**F**	**%**	**F**	**%**	**F**	**%**	***P*-value** ^a^
**Women’s education (years)**							**0.011**
None	10 455	64.9	5428	63.9	5027	66.0	
Primary (1–5 years)	1716	10.6	917	10.8	799	10.5	
Lower-secondary or higher (≥6 years)	3944	24.5	2156	25.4	1788	23.5	
**Women’s age at marriage (years)**							0.486
≤14 years	5624	34.9	2926	34.4	2698	35.4	
15 years	4153	25.8	2224	26.2	1929	25.3	
16–17 years	4670	29.0	2476	29.1	2194	28.8	
≥18 years	1668	10.4	875	10.3	793	10.4	
**Women’s height (cm) (*n *=* *12** **495)**							**0.038**
≤144.9 cm	1926	15.4	979	14.8	947	16.2	
145–154.9 cm	8070	64.6	4287	64.6	3783	64.5	
≥155 cm	2499	20.0	1367	20.6	1132	19.3	
**Parity (no. of births)**							**0.001**
0	5815	36.1	2999	35.3	2816	37.0	
1	4340	26.9	2261	26.6	2079	27.3	
2	3126	19.4	1653	19.4	1473	19.3	
≥3	2834	17.6	1588	18.7	1246	16.4	
**Husband’s education (years)**							**0.036**
None	7949	49.3	4119	48.5	3830	50.3	
Primary (1–5 years)	1860	11.5	978	11.5	882	11.6	
Lower-secondary or higher (≥6 years)	6306	39.1	3404	40.0	2902	38.1	
**Caste affiliation**							0.147
Disadvantaged: Muslim	3140	19.5	1619	19.0	1521	20.0	
Disadvantaged: Dalit	2557	15.9	1323	15.6	1234	16.2	
Middle: Janjati, Terai castes	6887	42.7	3651	42.9	3236	42.5	
Advantaged: Yadav, Brahmin	3531	21.9	1908	22.4	1623	21.3	
**Household assets**							**0.006**
1: Poorest	4013	24.9	2040	24.0	1973	25.9	
2	4053	25.1	2116	24.9	1937	25.4	
3	4036	25.0	2153	25.3	1883	24.7	
4: Richest	4013	24.9	2192	25.8	1821	23.9	
**Land-holding**							**0.011**
None	5830	36.2	2980	35.1	2850	37.4	
≤0.5 hectares	5230	32.5	2783	32.7	2447	32.1	
0.51–0.99 hectares	2343	14.5	1279	15.0	1064	14.0	
≥1 hectare	2712	16.8	1459	17.2	1253	16.5	
**Accessibility to big bazaar**							0.806
≤30 min	7996	49.6	4219	49.6	3777	49.6	
31–89 min	6605	41.0	3495	41.1	3110	40.8	
≥90 min	1514	9.4	787	9.3	727	9.5	

IQR, interquartile range; F, frequency.

P-values in bold indicate significance <0.05.

a Chi-squared test.

Compared to boys, girls were more likely to come from lower capital mothers and households with lower parity and shorter mothers, uneducated parents, poorer and low land-holding households (Table 1). There were no differences by infant sex in maternal age, age at marriage or accessibility to bazaar.

**Figure 2.**
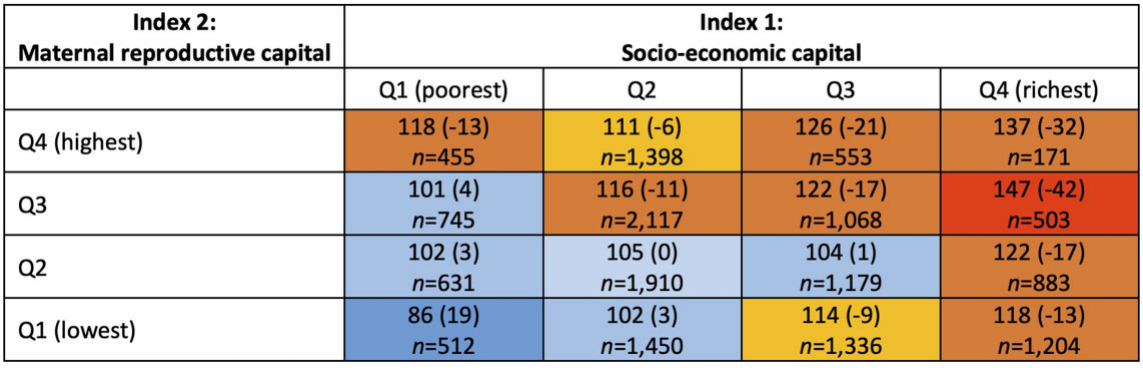
Heatmap of LBWSAT SRB by maternal capital composite indices 1 and 2 and excess girls or boys compared to global population SRB. The *X* axis is four groups of the first index and the *Y* axis is four groups of the second index. Each cell shows the SRB, Sex Ratio at Birth (males:females) in corresponding quartiles. In brackets, positive values = ‘excess daughters’ and negative values = ‘missing daughters’ relative to 100 sons. The number, *n*, in each cell is also shown. Differences in SRB between cells different by chi-squared test *P* < 0.001

The LBWSAT SRB by the two composite indices compared to global population SRB are shown in a heatmap (Fig. 2). In brackets, positive values indicated an excess of daughters and negative values indicated missing daughters relative to 100 sons. In each case, a lower quartile value indicated less capital, and these were the households and mothers to which girls were more likely to be born.

Mixed-effects logistic regression models testing the association of each individual factor with the odds of having a girl are shown in Supplementary Table S3. The odds of having a girl were higher for primigravidae, early marrying and shorter mothers, and for uneducated parents, disadvantaged castes, poorer and landless households. Accessibility to bazaar was not associated with the odds of having a girl.

Multivariable mixed-effects logistic regression models showed that lower parity, uneducated and poor women had a greater likelihood of having a girl (Table 2, Model 1). Independent of these factors, shorter maternal stature, which may be a developmental marker, and marrying early were also associated with a greater likelihood of having a girl (Model 2).

**Table 2.** Factors associated with the likelihood of having a girl

	**Model 1: Full sample**		**Model 2: Sub-sample with maternal height**	
	***n* = 16 115** ^a^		**n = 12 495** ^b^	
	**Conditional *R*^2^ = 0.006**		**Conditional *R*^2^ = 0.007**	
	**aOR (95% CI)**	***P*-value**	**aOR (95% CI)**	***P*-value**
**Women’s age (years)**	1.00 (0.99, 1.01)	0.989	1.00 (0.99, 1.01)	0.911
**Parity (no. of births)**				
0	1.31 (1.14, 1.49)	**<0.001**	1.30 (1.11, 1.51)	**0.001**
1	1.25 (1.11, 1.40)	**<0.001**	1.27 (1.11, 1.45)	**<0.001**
2	1.17 (1.05, 1.31)	**0.005**	1.18 (1.04, 1.33)	**0.009**
≥3 (ref)	1.00		1.00	
**Women’s education (years)**				
None	1.10 (1.00, 1.21)	**0.047**	1.12 (1.00, 1.24)	**0.043**
Primary (1–5 years)	1.04 (0.92, 1.17)	0.556	1.08 (0.95, 1.24)	0.240
≥Lower-secondary (≥6 years) (ref)	1.00		1.00	
**Women’s age at marriage (years)**				
≤14 years	1.07 (0.99, 1.14)	0.076	1.09 (1.01, 1.18)	**0.036**
≥15 years (ref)	1.00		1.00	
**Husband’s education (years)**				
None	1.01 (0.93, 1.10)	0.801	0.98 (0.90, 1.08)	0.739
Primary (1–5 years)	1.01 (0.90, 1.12)	0.896	1.00 (0.89, 1.14)	0.976
≥Lower-secondary (≥6 years) (ref)	1.00		1.00	
**Caste affiliation**				
Disadvantaged: Muslim	1.04 (0.93, 1.16)	0.515	0.99 (0.87, 1.12)	0.824
Disadvantaged: Dalit	1.05 (0.94, 1.18)	0.405	1.04 (0.91, 1.18)	0.575
Middle: Janjati, Terai castes	1.03 (0.94, 1.12)	0.534	1.00 (0.91, 1.10)	0.980
Advantaged: Yadav, Brahmin (ref)	1.00		1.00	
**Household assets**				
1: Poorest	1.12 (1.00, 1.24)	**0.041**	1.17 (1.04, 1.32)	**0.009**
2	1.07 (0.97, 1.18)	0.176	1.11 (0.99, 1.23)	0.073
3	1.03 (0.94, 1.13)	0.467	1.07 (0.97, 1.19)	0.196
4: Richest (ref)	1.00		1.00	
**Land-holding**				
None	1.02 (0.91, 1.14)	0.784	0.99 (0.87, 1.12)	0.857
≤0.5 hectares	0.97 (0.88, 1.07)	0.564	0.95 (0.85, 1.06)	0.377
0.51–0.99 hectares	0.95 (0.84, 1.06)	0.334	0.96 (0.84, 1.09)	0.492
≥1 hectare (ref)	1.00		1.00	
**Accessibility to big bazaar**				
≤30 min (ref)	1.00		1.00	
31–89 min	1.00 (0.93, 1.07)	0.897	1.00 (0.92, 1.08)	0.987
≥90 min	1.02 (0.90, 1.14)	0.777	1.08 (0.94, 1.23)	0.287
**Maternal height (cm)**				
≤144.9 cm			1.15 (1.02, 1.29)	**0.027**
145–154.9 cm			1.06 (0.97, 1.16)	0.214
≥155 cm (ref)			1.00	
Intercept	0.62 (0.45, 0.85)	0.003	0.57 (0.39, 0.81)	0.002

aOR, adjusted odds ratio; 95% CI, 95% confidence interval.

P-values in bold indicate significance <0.05.

a *n *=* *8501 boys versus *n *=* *7614 girls.

b *n *=* *6633 boys versus *n *=* *5862 girls. Mixed-effects logistic regression models include fixed and random effects estimates for geographic clusters and control for trial arm. As associations of trial arm with the odds of having a girl were not statistically significant, they are not reported in the table.

The independent association of composite maternal index 1 (socio-economic capital) and composite maternal index 2 (reproductive capital) with the odds of having a girl are shown in Table 3. Mothers with the lowest level of socio-economic and reproductive capital had a greater likelihood of having a girl.

**Table 3.** Association of two composite indices of the capacity for parental investment with the likelihood of having a girl

	***n *=* *16 082** ^a^	
	**Conditional *R*^2^ = 0.005**	
	**aOR (95% CI)**	***P*-value**
**Composite index 1**: **socio-economic capital^b^**		
1: Low	1.29 (1.15, 1.44)	**<0.001**
2	1.19 (1.08, 1.30)	**<0.001**
3	1.12 (1.02, 1.24)	**0.023**
4: High (ref)	1.00	
**Composite index 2: maternal reproductive capital**		
1: Low	1.15 (1.04, 1.27)	**0.006**
2	1.13 (1.02, 1.24)	**0.018**
3	1.01 (0.91, 1.11)	0.885
4: High (ref)	1.00	
Intercept	0.71 (0.62, 0.81)	<0.001

aOR, adjusted odds ratio; 95% CI, 95% confidence interval.

a *n* = 8501 boys versus *n *=* *7614 girls.

b Composite index 1: husband’s education, assets, maternal education, land and caste.

3 Composite index 2: maternal parity and age. Mixed-effects logistic regression models include fixed and random effects estimates for geographic clusters and control for trial arm. As associations of trial arm with the odds of having a girl were not statistically significant, they are not reported in the table.

P-values in bold indicate significance <0.05.

## DISCUSSION

We investigated whether girls and boys have an equal chance of being born into households with a greater capacity to invest in their offspring. Our first key finding was that the SRB was 112:100. Relative to the United Nations (UN) value of 105:100 as the ‘expected SRB’ [2], this indicates a loss of 7 girls for every 100 boys. Although our study suffered from missing data, our calculations suggest that this is very unlikely to account for the observed SRB. High SRBs achieved through sex selection have been explained by Guilmoto through a ‘ready, willing and able’ framework [66], whereby families may be ready because they have a preference for sons, willing because of cultural acceptability of using prenatal sex selection of foetuses and able if they can access the appropriate technology for both foetal sex determination and abortion. This cultural manipulation of the SRB reflects a ‘fertility squeeze’ produced by declining fertility.

The overall SRB of 112 in our study was higher than values of 107 reported previously in Nepal, 110 in India and 109 in southern Asia [4]. However, this aggregate ratio masks substantial geographic variation across and within countries, and associations with parity. For example, a study of different communities in Bangladesh, Pakistan, India and Nepal estimated SRBs ranging from 102 to 128 [67]. The Nepal component of this study found that two adjoining hill districts in the western development region had SRBs of 114 and 102 [26]. Using 2016 DHS data, Channon *et al*. [5] estimated an overall SRB of 106, which was lower than our value, but also a conditional SRB of 115 for second births when the first-born was female. However, an estimation of DHS data from 2012–16 found an overall SRB of 111, which is closer to our study [29]. A high SRB of 112 was also found in a study of ∼9300 hospital births in Palpa District, southwest Nepal (2008–15) [68], while analysis of ∼75 000 births (2015–16) across six tertiary hospitals found an SRB of 117 [31].

Our second key finding, consistent with the first, is that girls were disproportionately born to mothers with reduced capacity to invest in them. This was evident both for individual markers of capital, such as lack of education, early marriage, shorter stature and poor households, and also for the two composite markers indexing maternal socio-economic and reproductive capital. We also found the highest SRB values in the families with the greatest socio-economic capital, suggesting that these families are likely to be actively aborting female foetuses, resulting in live births being strongly skewed towards boys. We found no indication that proximity to large bazaars predicted the likelihood of mothers having a girl, hence parental education and wealth are likely to have been the primary driving factors of such sex selection. It is also possible that the necessary technology is not available in all places with big shops.

Conversely, SRBs below 105 are likely to reflect both biological factors and the lack of access and means to pay for sex-diagnostic and abortion facilities. It is unlikely that the poorest families would be deliberately aiming to increase the likelihood of having daughters. First, they are least likely to have access to foetal sex detection and abortion facilities. Second, it is unlikely they would have an active preference for daughters, given the dowry costs associated with them, and the ‘missed opportunity’ to have a son who could contribute economically to the care of parents in old age. Therefore, we assume that the higher likelihood of daughters among families with lower reproductive and socio-economic capital, indicated by SRB values <105, indicates a contribution of biological mechanisms. This is particularly the case for the association of SRB with reproductive capital, where amongst families in the bottom quartile for socio-economic capital, there was a dose–response increasing the likelihood of having a girl. The one exception, where even poor families might seek to influence the sex of the child, is where family size is already high. This may explain the SRB of 118 in the lowest socio-economic quartile and the highest reproductive capital quartile. If poor families lack access to foetal sex detection and abortion facilities, then this may be achieved through infanticide, which was a commonly used method of birth control in some parts of Asia in the past [69]. However, evidence for infanticide in contemporary Nepal is inconsistent [70, 71].

Overall, our results indicate the differential use of sex-determining technology in association with family size, alongside girls being more likely to be born at lower parities and into larger, poorer and less educated families. Although we focused on the factors associated with a greater likelihood of female births, our findings are consistent with other studies investigating the factors associated with an elevated SRB or a greater likelihood of male births [33, 34]. For example, similar to our finding that poor uneducated mothers were more likely to bear daughters, other studies have found that boys are more likely to be born into richer and more educated families [5, 26, 28, 31, 67, 68]. Underlying factors linked with skewed SRB values in these studies likewise include persistent son preference, use of sex-determination technology and lower desired fertility [5, 17, 29, 66]. In Nepal, policy also matters, with the legalization of abortion (although not sex-determination technology) and a lack of community-based advocacy programmes promoting the value of girls associated with higher SRBs in some regions [26, 28, 67].

We have previously shown that maternal BMI rises with age and parity in this population, which might increase the likelihood of sons being born when mothers have higher reproductive capital [59]. A study in rural Ethiopia found similar results: compared to women in the lowest quartile of fat and muscle mass, those in the upper quartile were twice as likely to have a son [72]. Another possible contributing factor is greater male foetal mortality or stillbirths among families with lower socio-economic and reproductive capital, as modelled by the ‘frail male’ conceptual framework [15]. However, we did not have the data to address that issue. We also did not have any physiological data to explore what biological mechanisms may contribute to these associations, and hence our analysis cannot be considered a test of the Trivers–Willard hypothesis, which aimed to evaluate SRB variability in terms of its implications for maternal lifetime reproductive fitness [7]. Rather, our focus here was on the implications for daughters of being differentially born into families with a lower capacity to invest in them.

That girls are more likely to occupy a ‘lower capital niche’ in early life is of evolutionary importance because this exposure is associated with adverse adult health and human capital outcomes, and lower reproductive fitness [20–23]. We have previously shown in a Brazilian birth cohort that mothers with low physical and socio-economic capital have daughters that are themselves more likely to reproduce early, drop out of school, and be shorter in adult life [73]. Although low maternal capital may also impact sons, these effects would be reduced if on average in the population, sons were born to higher capital mothers compared to daughters.

Our findings have implications for understanding the inter-generational transmission of gender inequality [74]. The patterns we have demonstrated may contribute to the inter-generational propagation of gender inequality in this population, whereby less educated daughters marry at a younger age and into lower socio-economic status households, and then are more likely to give birth to daughters themselves who are exposed to the same developmental niche. A key issue is that even if parents do not express any gender bias, and would inherently invest equally in boys and girls after birth, the patterns we show here will still replicate gender inequality. For example, if both wealthy and poor families placed equal emphasis on taking girls and boys to a doctor, but if compared to boys, girls were relatively concentrated in poorer families who were less able to afford doctors, then overall in the population fewer girls than boys would be seen by doctors. Girls are also more likely to be born into larger families and at earlier parities, which can have consequences for their future prospects if families are either unwilling or unable to invest equally in each child [34]. These issues are likely to be important in Nepal, which ranks 110 out of 189 countries on the Gender Inequality Index, a composite measure reflecting inequality in achievement between women and men in reproductive health, empowerment and the labour market [75].

### Strengths and limitations

Our study has some strengths, including the large sample size in a disadvantaged rural population, and the availability of data on a wide range of maternal and household characteristics.

The study also has several limitations. First, there were missing data on both offspring sex, and on maternal predictors. However, we think these are unlikely to bias our findings. Families with missing data on sex would have to have very strong bias to daughters to negate the observed overall SRB of 112. Similarly, differences between mothers with or lacking offspring sex in socio-economic variables were very modest, and again could only bias our findings if the associations of between these variables and sex were completely different from those observed in the main sample. There was also no difference in SRB between mothers included in the analysis versus excluded due to missing predictor data. Finally, the overall SRB is similar to that reported in other studies in Nepal. Overall, these patterns in combination with our simulations suggest that missing data are very unlikely to account for the patterns we observe.

Second, we have no direct information on foetal sex detection and abortion, and our interpretation of the data has to assume that these technologies and facilities are available and in demand in this population. This is similar to other studies in the region [5].

Third, given that we used all available socio-economic and reproductive traits in our PCA analysis, we could control only for confounders relating to the study design. There may be confounding factors that we did not measure that could contribute to the associations. Fourth, we cannot evaluate whether males had higher foetal mortality (and stillbirth) in lower socio-economic households, which could result in the higher concentration of female births. Fifth, we could not explore whether the sex of previous siblings increased the likelihood of having a girl, because not every participant answered this question.

Finally, our results apply to a relatively disadvantaged society with patrilineal patrilocal family structure, where son preference is strong and dowry payments for daughters present economic challenges. Our findings may not be generalizable outside the study context and similar populations.

## CONCLUSIONS

Amartya Sen termed the global shortfall of women, from not only the expected number at birth but also throughout the life-course, as ‘missing women’ [76]. Arguably, not being given the chance to be born at all is the greatest form of discrimination against girls, and the SRB of 112 in our study suggests that this issue is evident in rural lowland Nepal. However, where son preference is high, some girls born are ‘unwanted’, and are likely to face a range of risks, which in some societies can include premature mortality arising from female infanticide and maltreatment and neglect during early childhood, adolescence and adulthood across a range of dimensions [24, 38, 39, 77]. We have added to this debate, by showing that in this population, on average girls are more likely than boys to be born into families with a lower capacity to invest in them.

Our results show that girls do indeed start life facing composite disadvantages compared to boys, being more likely than boys to be born to mothers with lower socio-economic status and lower reproductive capital. Such households are likely to have fewer resources to educate, feed and adequately care for all children. This may be compounded by the fact that in societies where sons are preferred, girls may not receive their fair share of support. This cycle of disadvantage and sex discrimination may then continue into the next generation.

## SUPPLEMENTARY DATA

Supplementary data is available at *EMPH* online.

## Supplementary Material

eoac029_Supplementary_Data

## Data Availability

Requests to access the dataset, through a data sharing agreement, should be directed to Dr Naomi Saville, n.saville@ucl.ac.uk.
