# Supplementary material for: Girls start life on an uneven playing field: Evidence from lowland rural Nepal
Source: Evol Med Public Health. 2022 Aug 4;10(1):339–51. doi: 10.1093/emph/eoac029 (PMC9384836; doi:10.1093/emph/eoac029)
Supplement: eoac029_Supplementary_Data [file eoac029_supplementary_data.docx]

**Girls start life on an uneven playing field: Evidence from lowland rural Nepal**

**SUPPLEMENTARY MATERIALS**

**Figure S1.** Biplot of PCA indexing maternal socio-economic and reproductive capital components

**Figure S2.** Scatter plot showing how sex ratio in total sample would manifest, depending on the sex ratio in subsample missing data on sex

**Table S1.** Bias in missing data on child sex

**Table S2**. Bias in child sex

**Table S3.** Mixed-effects logistic regression models investigating associations of individual

factors associated with the likelihood of having a girl

**Figure S1. Biplot of PCA indexing maternal reproductive capital and socio-economic capital components**

capital

Maternal reproductive

Socio-economic capital

**Figure S2. Scatter plot showing how sex ratio in total sample would manifest, depending on the sex ratio in subsample missing data on neonatal sex (n=5,731).** The sex ratio in the sample missing such data (A) would need to be 0.84, compared with 1.12 in the much larger sample providing such data (B, n=18,951), to produce the UN expected ‘natural’ ratio of 1.05 for the total sample (A+B, n=24,682).


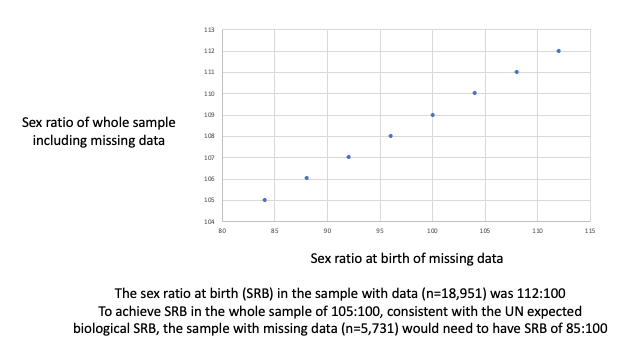


**Table S1. Bias in missing data on child sex**

|  | **Have data on child sex**  **(*n*=18,951)** | | **Missing data on child sex**  **(*n*=5,731)** | | ***p*-value**^a^ |
| --- | --- | --- | --- | --- | --- |
|  | **Median** | **IQR** | **Median** | **IQR** |  |
| **Women’s age (y)** | 21 | 6 | 20 | 5 | **0.001** |
|  | **F** | **%** | **F** | **%** | ***p*-value**^b^ |
| **Women’s education (y)** |  |  |  |  | **0.001** |
| None | 12,365 | 65.5 | 2,863 | 58.8 |  |
| Primary (1-5 years) | 1,927 | 10.2 | 524 | 10.8 |  |
| Lower-secondary or higher (≥6 years) | 4,600 | 24.3 | 1,481 | 30.4 |  |
| **Women’s marriage age (y)** |  |  |  |  | **0.001** |
| ≤14 years | 5,703 | 35.0 | 1,401 | 29.5 |  |
| 15 years | 4,181 | 25.7 | 1,217 | 25.6 |  |
| 16-17 years | 4,718 | 29.0 | 1,500 | 31.6 |  |
| ≥18 years | 1,684 | 10.3 | 629 | 13.3 |  |
| **Women’s height (cm)** |  |  |  |  | 0.295 |
| ≤144.9 cm | 2,257 | 15.4 | 260 | 16.8 |  |
| 145-154.9 cm | 9,504 | 64.7 | 994 | 64.1 |  |
| ≥155 cm | 2,938 | 20.0 | 296 | 19.1 |  |
| **Parity (no. of births)** |  |  |  |  | **0.001** |
| 0 | 6,679 | 35.5 | 2,100 | 44.4 |  |
| 1 | 5,074 | 26.9 | 1,293 | 27.3 |  |
| 2 | 3,702 | 19.7 | 745 | 15.7 |  |
| ≥3 | 3,373 | 17.9 | 594 | 12.6 |  |
| **Husband’s education (y)** |  |  |  |  | **0.001** |
| None | 9,242 | 48.9 | 2,197 | 45.1 |  |
| Primary (1-5 years) | 2,180 | 11.5 | 557 | 11.4 |  |
| Lower-secondary or higher (≥ 6years) | 7,470 | 39.5 | 2,113 | 43.1 |  |
| **Caste affiliation** (*n*=5,695) |  |  |  |  | **0.001** |
| Disadvantaged: Muslim | 3,678 | 19.4 | 1,029 | 18.1 |  |
| Disadvantaged: Dalit | 3,064 | 16.2 | 1,041 | 18.3 |  |
| Middle: Janjati, Terai castes | 8,078 | 42.6 | 2,246 | 39.4 |  |
| Advantaged: Yadav, Brahmin | 4,131 | 21.8 | 1,379 | 24.2 |  |
| **Household assets** |  |  |  |  | **0.001** |
| 1: Poorest | 4,677 | 25.0 | 1,109 | 23.2 |  |
| 2 | 4,612 | 24.7 | 1,101 | 23.0 |  |
| 3 | 4,671 | 25.0 | 1,242 | 26.0 |  |
| 4: Richest | 4,716 | 25.3 | 1,329 | 27.8 |  |
| **Land-holding** |  |  |  |  | **0.001** |
| None | 6,775 | 39.7 | 1,680 | 35.8 |  |
| ≤0.5 hectares | 5,231 | 30.7 | 1,393 | 29.7 |  |
| 0.51-0.99 hectares | 2,344 | 13.7 | 739 | 15.8 |  |
| ≥1 hectare | 2,712 | 15.9 | 876 | 18.7 |  |
| **Access to big bazaar** |  |  |  |  | 0.108 |
| ≤30 minutes | 7,997 | 49.6 | 2,271 | 49.5 |  |
| 31-89 minutes | 6,607 | 41.0 | 1,931 | 42.1 |  |
| ≥90 minutes | 1,514 | 9.4 | 388 | 8.5 |  |

IQR, Interquartile range. F, frequency. %, percentage. ^a^Kruskal-Wallis Test. ^b^Chi squared test.

**Table S2. Bias in child sex**

|  | **Sample used in analysis**  **(*n*=16,115)** | | **Excluded for missing data on key predictor variables**  **(*n*=2,836)** | |  |
| --- | --- | --- | --- | --- | --- |
|  | **F** | **%** | **F** | **%** | ***p*-value**^a^ |
| **Child sex** |  |  |  |  | 0.446 |
| Boys | 8,501 | 52.8 | 1,518 | 53.5 |  |
| Girls | 7,614 | 47.2 | 1,318 | 46.5 |  |
|  | **Ratio** | | **Ratio** | |  |
| **Sex ratio** | 111.6 | | 115.2 | |  |

F, frequency. %, percentage. ^a^Chi squared test.

**Table S3. Mixed-effects logistic regression models investigating associations of individual factors associated with the likelihood of having a girl**

| **Variable** | **aOR (95%CI)** | ***p*-value** |
| --- | --- | --- |
| **Model 1: Women’s age (y)**^a^ *n*=16,115 Conditional *R^2^* 0.004 | 0.99 (0.98, 0.99) | **0.005** |
| Intercept | 1.08 (0.92, 1.28) | 0.338 |
| **Model 2: Parity (no. of births)**^a^ *n*=16,115 Conditional *R^2^* 0.005 |  |  |
| 0 | 1.21 (1.10, 1.32) | **0.001** |
| 1 | 1.81 (1.07, 1.30) | **0.001** |
| 2 | 1.14 (1.03, 1.27) | **0.011** |
| ≥3 (ref) | 1.00 |  |
| Intercept | 0.77 (0.69, 0.85) | 0.001 |
| **Model 3: Women’s age and parity**^a^ *n*=16,115 Conditional *R^2^* 0.005 |  |  |
| Women’s age (y) | 1.00 (0.99, 1.01) | 0.918 |
| Parity (no. of births) |  |  |
| 0 | 1.21 (1.07, 1.38) | **0.003** |
| 1 | 1.18 (1.06, 1.33) | **0.004** |
| 2 | 1.14 (1.03, 1.28) | **0.016** |
| ≥3 (ref) |  |  |
| Intercept | 0.76 (0.57, 1.01) | 0.055 |
| **Model 4: Women’s education (y)**^a^ *n*=16,115 Conditional *R^2^* 0.006 |  |  |
| None | 1.17 (1.09, 1.27) | **0.001** |
| Primary (1-5 years) | 1.07 (0.95, 1.20) | 0.245 |
| Lower-secondary or higher (≥6 years) (ref) | 1.00 |  |
| Women’s age (y) | 1.00 (0.99, 1.01) | 0.715 |
| Parity (no. of births) |  |  |
| 0 | 1.25 (1.10, 1.43) | **0.001** |
| 1 | 1.21 (1.08, 1.36) | **0.001** |
| 2 | 1.15 (1.03, 1.28) | **0.012** |
| ≥3 (ref) | 1.00 |  |
| Intercept | 0.69 (0.52, 0.93) | 0.014 |
| **Model 5: Women’s age at marriage (y)**^a^ *n*=16,115 Conditional *R^2^* 0.005 |  |  |
| ≤14 years | 1.09 (1.01, 1.17) | **0.018** |
| ≥15 years (ref) | 1.00 |  |
| Women’s age (y) | 1.00 (0.99, 1.01) | 0.666 |
| Parity (no. of births) |  |  |
| 0 | 1.27 (1.11, 1.44) | **0.001** |
| 1 | 1.22 (1.08, 1.37) | **0.001** |
| 2 | 1.16 (1.04, 1.30) | **0.008** |
| ≥3 (ref) | 1.00 |  |
| Intercept | 0.69 (0.51, 0.93) | 0.015 |
| **Model 6: Husband’s education (y)**^a^ *n*=16,115 Conditional *R^2^* 0.005 |  |  |
| None | 1.12 (1.04, 1.19) | **0.002** |
| Primary (1-5 years) | 1.07 (0.97, 1.19) | 0.189 |
| Lower-secondary or higher (≥6years) (ref) | 1.00 |  |
| Women’s age (y) | 1.00 (0.99, 1.01) | 0.909 |
| Parity (no. of births) |  |  |
| 0 | 1.23 (1.08, 1.40) | **0.001** |
| 1 | 1.20 (1.07, 1.35) | **0.002** |
| 2 | 1.15 (1.03, 1.29) | **0.011** |
| ≥3 (ref) | 1.00 |  |
| Intercept | 0.72 (0.54, 0.96) | 0.025 |
| **Model 7: Caste affiliation**^a^ *n*=16,115 Conditional *R^2^* 0.005 |  |  |
| Disadvantaged: Muslim | 1.12 (1.01, 1.23) | **0.025** |
| Disadvantaged: Dalit | 1.10 (0.99, 1.22) | 0.082 |
| Middle: Janjati, Terai castes | 1.04 (0.95, 1.13) | 0.384 |
| Advantaged: Yadav, Brahmin (ref) | 1.00 |  |
| Women’s age (y) | 1.00 (0.99, 1.01) | 0.868 |
| Parity (no. of births) |  |  |
| 0 | 1.23 (1.08, 1.40) | **0.002** |
| 1 | 1.20 (1.07, 1.35) | **0.002** |
| 2 | 1.15 (1.03, 1.29) | **0.010** |
| ≥3 (ref) | 1.00 |  |
| Intercept | 0.70 (0.52, 0.94) | 0.019 |
| **Model 8: Household assets**^a^ *n*=16,115 Conditional *R^2^* 0.006 |  |  |
| 1: Poorest | 1.19 (1.09, 1.31) | **0.001** |
| 2 | 1.12 (1.02, 1.22) | **0.015** |
| 3 | 1.06 (0.97, 1.16) | 0.208 |
| 4: Richest (ref) | 1.00 |  |
| Women’s age (y) | 1.00 (0.99, 1.01) | 0.979 |
| Parity (no. of births) |  |  |
| 0 | 1.23 (1.09, 1.40) | **0.001** |
| 1 | 1.20 (1.07, 1.35) | **0.002** |
| 2 | 1.15 (1.03, 1.28) | **0.011** |
| ≥3 (ref) | 1.00 |  |
| Intercept | 0.70 (0.52, 0.93) | 0.015 |
| **Model 9: Land holding**^a^ *n*=16,115 Conditional *R^2^* 0.005 |  |  |
| None | 1.13 (1.03, 1.24) | **0.009** |
| ≤0.5 hectares | 1.04 (0.94, 1.14) | 0.464 |
| 0.51-0.99 hectares | 0.97 (0.87, 1.09) | 0.632 |
| ≥1 hectare (ref) | 1.00 |  |
| Women’s age (y) | 1.00 (0.99, 1.01) | 0.933 |
| Parity (no. of births) |  |  |
| 0 | 1.23 (1.08, 1.40) | **0.002** |
| 1 | 1.20 (1.07, 1.35) | **0.002** |
| 2 | 1.15 (1.03, 1.29) | **0.010** |
| ≥3 (ref) | 1.00 |  |
| Intercept | 0.71 (0.53, 0.95) | 0.023 |
| **Model 10: Accessibility to big bazaar**^a^ *n*=16,115 Conditional *R^2^* 0.005 |  |  |
| ≤30 minutes (ref) | 1.00 |  |
| 31-89 minutes | 1.00 (0.94, 1.08) | 0.899 |
| ≥90 minutes | 1.04 (0.93, 1.17) | 0.493 |
| Women’s age (y) |  |  |
| Parity (no. of births) | 1.00 (0.99, 1.01) | 0.907 |
| 0 | 1.21 (1.07, 1.38) | **0.003** |
| 1 | 1.19 (1.06, 1.33) | **0.004** |
| 2 | 1.14 (1.03, 1.28) | **0.015** |
| ≥3 (ref) | 1.00 |  |
| Intercept | 0.75 (0.56, 1.00) | 0.050 |
| **Model 11: Maternal height (cm)** *n*=12,495^a^ Conditional *R^2^* 0.005 |  |  |
| ≤144.9 cm | 1.18 (1.05, 1.33) | **0.006** |
| 145-154.9cm | 1.07 (0.98, 1.17) | 0.129 |
| ≥155 cm (ref) | 1.00 |  |
| Women’s age (y) | 1.00 (0.99, 1.01) | 0.899 |
| Parity (no. of births) |  |  |
| 0 | 1.19 (1.03, 1.38) | **0.016** |
| 1 | 1.21 (1.06, 1.37) | **0.005** |
| 2 | 1.15 (1.02, 1.30) | **0.023** |
| ≥3 (ref) | 1.00 |  |
| Intercept | 0.69 (0.50, 0.97) | 0.031 |

*Notes:* aOR, adjusted Odds Ratio. CI, 95% Confidence Interval. ^a^n=8,501 boys vs. n=7,614 girls.  ^b^n=6,633 boys vs. n=5,862 girls. Models include fixed and random effects estimates for geographic clusters and control for trial arm. As associations of trial arm with the odds of having a girl were not statistically significant, they are not reported in the Table.
